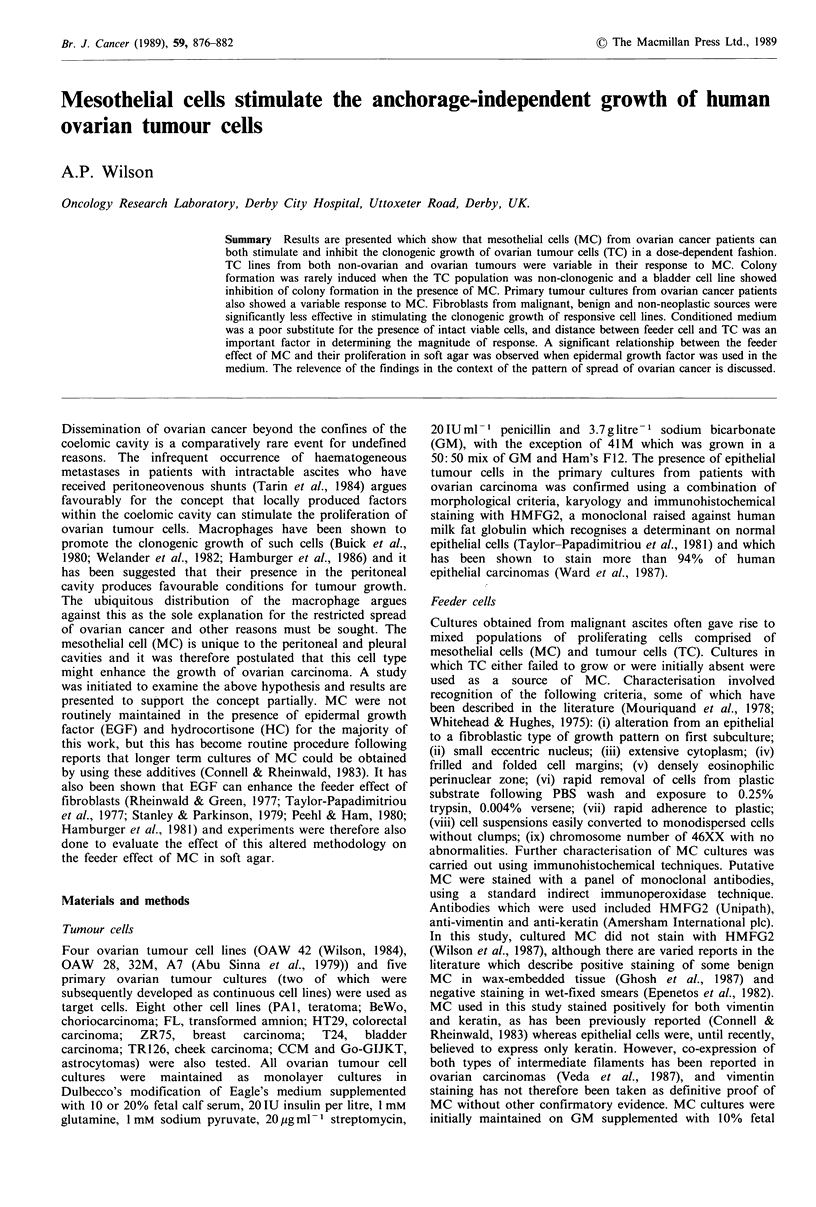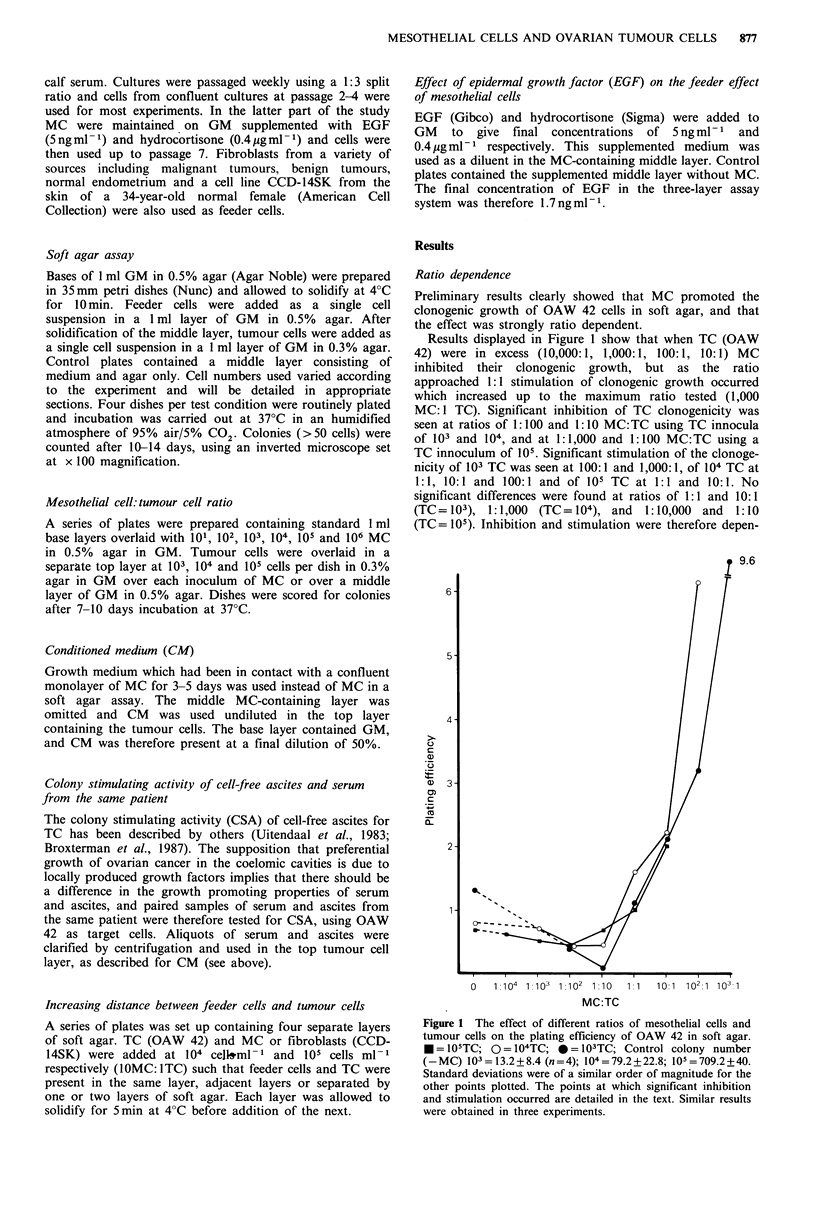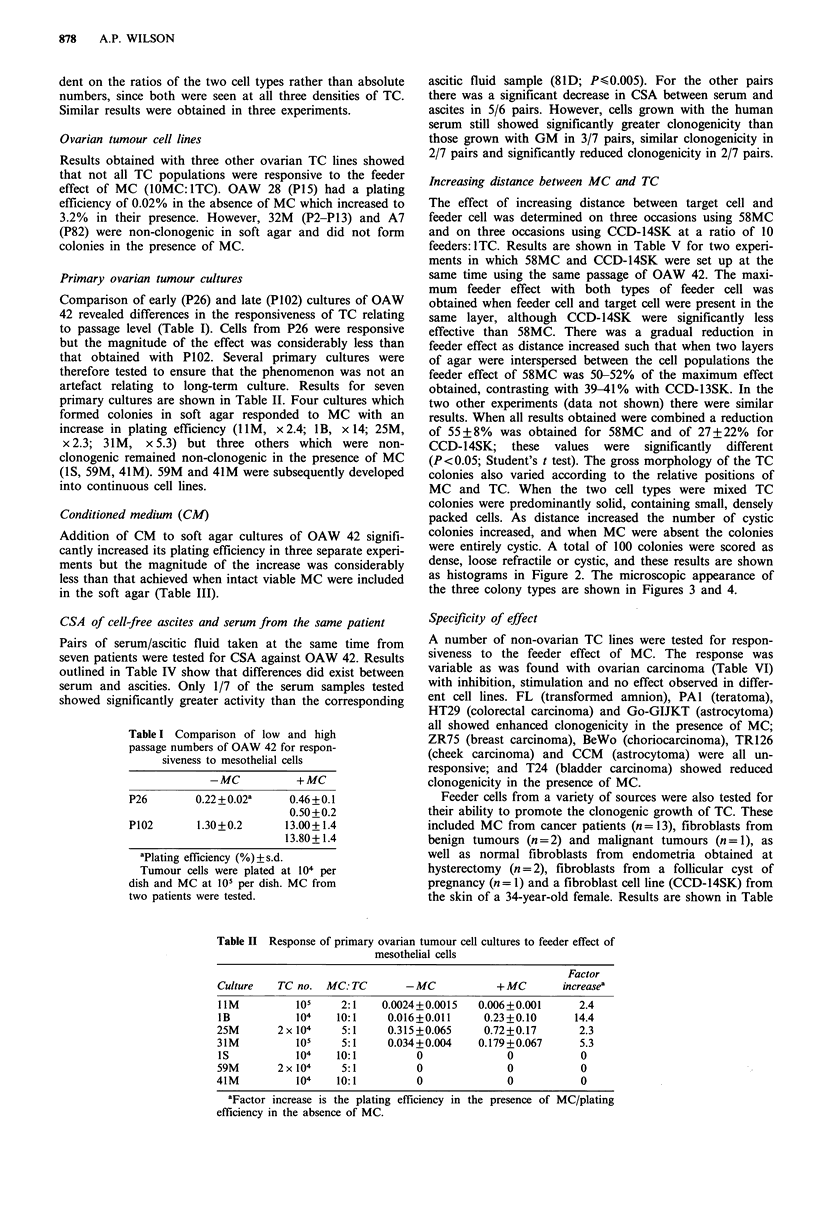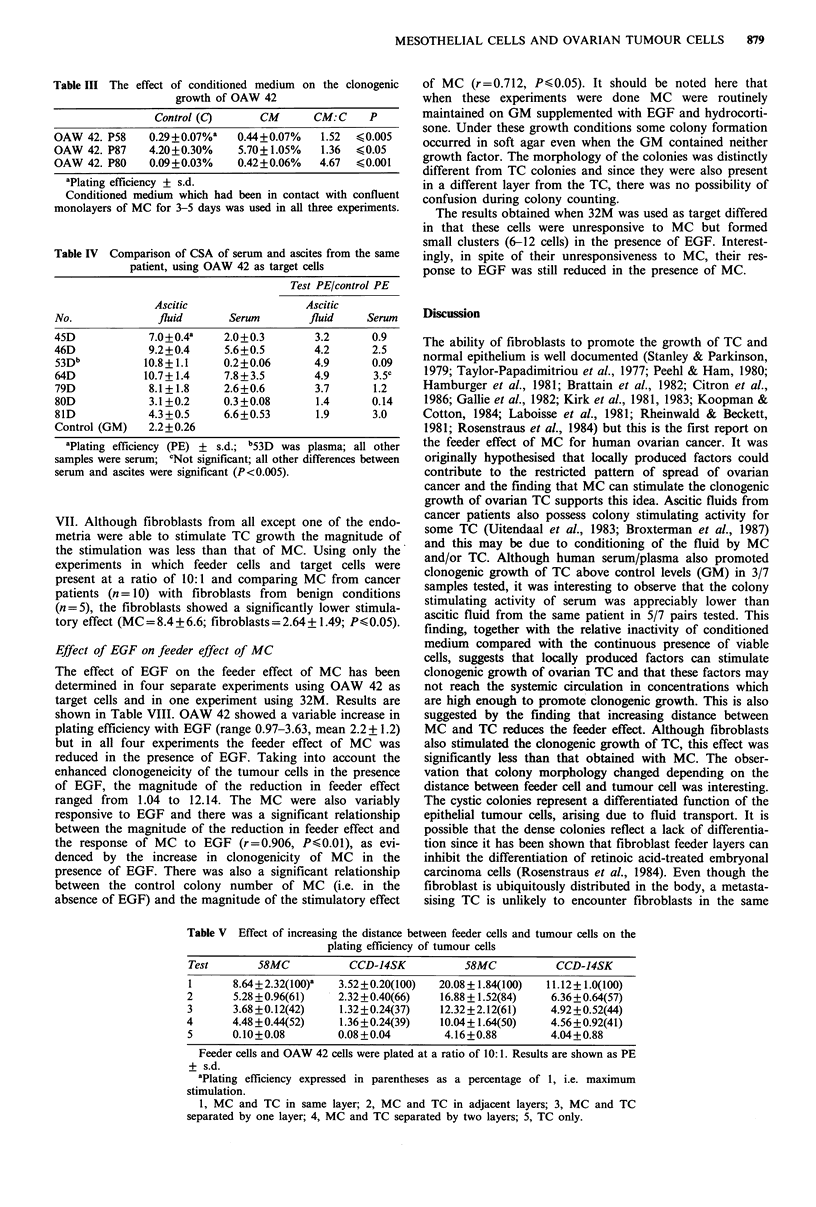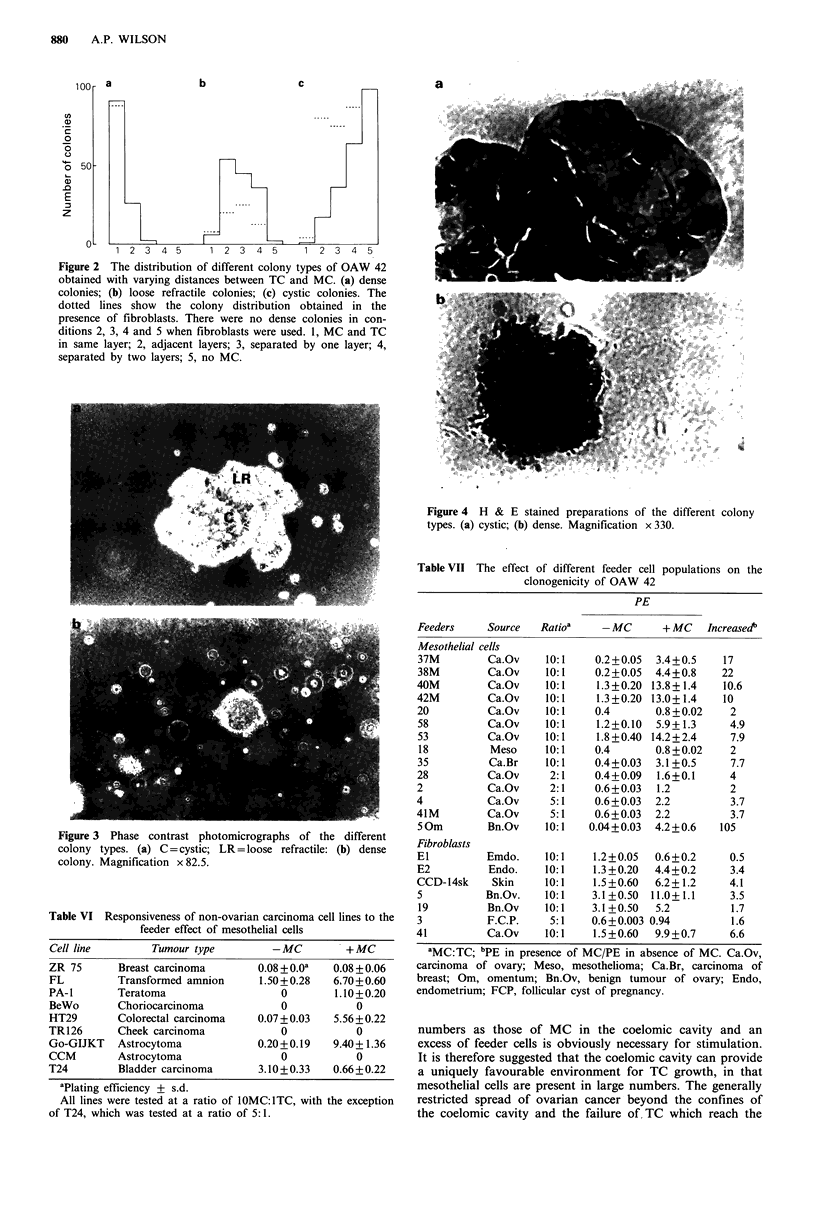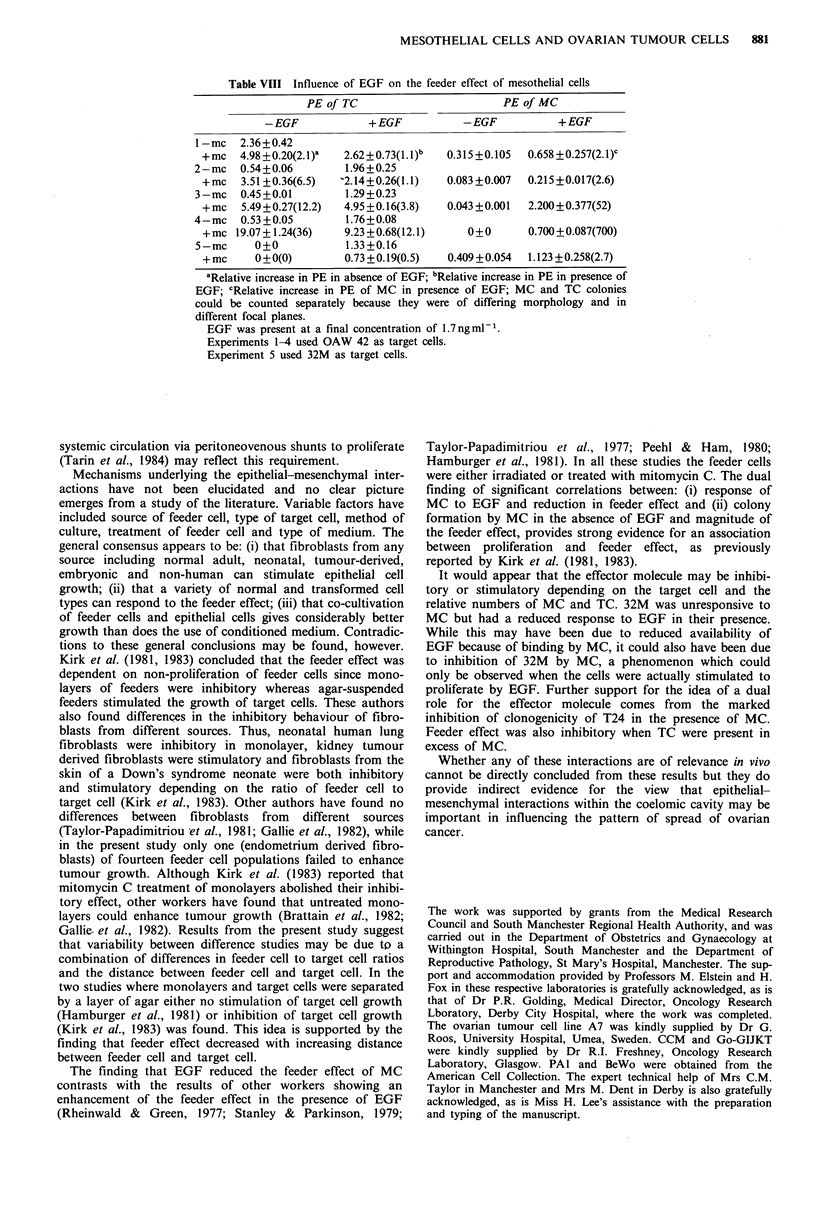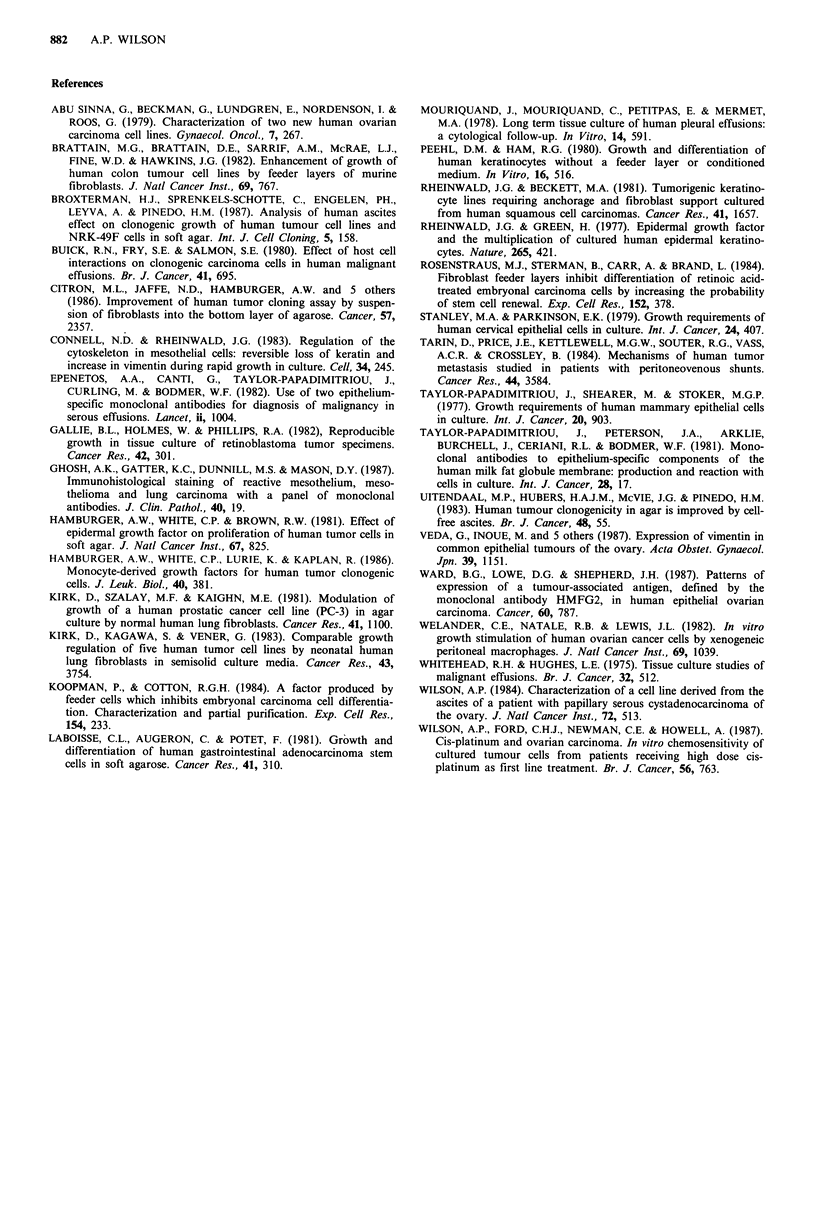# Mesothelial cells stimulate the anchorage-independent growth of human ovarian tumour cells.

**DOI:** 10.1038/bjc.1989.186

**Published:** 1989-06

**Authors:** A. P. Wilson

**Affiliations:** Oncology Research Laboratory, Derby City Hospital, UK.

## Abstract

**Images:**


Br. J. Cancer (1989), 59, 876–882 © The Macmillan Press Ltd., 1989

### Mesothelial cells stimulate the anchorage-independent growth of human ovarian tumour cells

A.P. Wilson

*Oncology Research Laboratory, Derby City Hospital, Uttoxeter Road, Derby, UK.*

**Summary** Results are presented which show that mesothelial cells (MC) from ovarian cancer patients can both stimulate and inhibit the clonogenic growth of ovarian tumour cells (TC) in a dose-dependent fashion. TC lines from both non-ovarian and ovarian tumours were variable in their response to MC. Colony formation was rarely induced when the TC population was non-clonogenic and a bladder cell line showed inhibition of colony formation in the presence of MC. Primary tumour cultures from ovarian cancer patients also showed a variable response to MC. Fibroblasts from malignant, benign and non-neoplastic sources were significantly less effective in stimulating the clonogenic growth of responsive cell lines. Conditioned medium was a poor substitute for the presence of intact viable cells, and distance between feeder cell and TC was an important factor in determining the magnitude of response. A significant relationship between the feeder effect of MC and their proliferation in soft agar was observed when epidermal growth factor was used in the medium. The relevence of the findings in the context of the pattern of spread of ovarian cancer is discussed.

Dissemination of ovarian cancer beyond the confines of the coelomic cavity is a comparatively rare event for undefined reasons. The infrequent occurrence of haematogeneous metastases in patients with intractable ascites who have received peritoneovenous shunts (Tarin *et al.*, 1984) argues favourably for the concept that locally produced factors within the coelomic cavity can stimulate the proliferation of ovarian tumour cells. Macrophages have been shown to promote the clonogenic growth of such cells (Buick *et al.*, 1980; Welander *et al.*, 1982; Hamburger *et al.*, 1986) and it has been suggested that their presence in the peritoneal cavity produces favourable conditions for tumour growth. The ubiquitous distribution of the macrophage argues against this as the sole explanation for the restricted spread of ovarian cancer and other reasons must be sought. The mesothelial cell (MC) is unique to the peritoneal and pleural cavities and it was therefore postulated that this cell type might enhance the growth of ovarian carcinoma. A study was initiated to examine the above hypothesis and results are presented to support the concept partially. MC were not routinely maintained in the presence of epidermal growth factor (EGF) and hydrocortisone (HC) for the majority of this work, but this has become routine procedure following reports that longer term cultures of MC could be obtained by using these additives (Connell & Rheinwald, 1983). It has also been shown that EGF can enhance the feeder effect of fibroblasts (Rheinwald & Green, 1977; Taylor-Papadimitriou *et al.*, 1977; Stanley & Parkinson, 1979; Peehl & Ham, 1980; Hamburger *et al.*, 1981) and experiments were therefore also done to evaluate the effect of this altered methodology on the feeder effect of MC in soft agar.

### Materials and methods

### *Tumour cells*

Four ovarian tumour cell lines (OAW 42 (Wilson, 1984), OAW 28, 32M, A7 (Abu Sinna *et al.*, 1979)) and five primary ovarian tumour cultures (two of which were subsequently developed as continuous cell lines) were used as target cells. Eight other cell lines (PA1, teratoma; BeWo, choriocarcinoma; FL, transformed amnion; HT29, colorectal carcinoma; ZR75, breast carcinoma; T24, bladder carcinoma; TR126, cheek carcinoma; CCM and Go-GIJKT, astrocytomas) were also tested. All ovarian tumour cell cultures were maintained as monolayer cultures in Dulbecco's modification of Eagle's medium supplemented with 10 or 20% fetal calf serum, 20 IU insulin per litre, 1 mM glutamine, 1 mM sodium pyruvate, 20 $\mu g\,ml^{-1}$ streptomycin, 20 $IU\,ml^{-1}$ penicillin and 3.7 $g\,litre^{-1}$ sodium bicarbonate (GM), with the exception of 41M which was grown in a 50:50 mix of GM and Ham's F12. The presence of epithelial tumour cells in the primary cultures from patients with ovarian carcinoma was confirmed using a combination of morphological criteria, karyology and immunohistochemical staining with HMFG2, a monoclonal raised against human milk fat globulin which recognises a determinant on normal epithelial cells (Taylor–Papadimitriou *et al.*, 1981) and which has been shown to stain more than 94% of human epithelial carcinomas (Ward *et al.*, 1987).

### *Feeder cells*

Cultures obtained from malignant ascites often gave rise to mixed populations of proliferating cells comprised of mesothelial cells (MC) and tumour cells (TC). Cultures in which TC either failed to grow or were initially absent were used as a source of MC. Characterisation involved recognition of the following criteria, some of which have been described in the literature (Mouriquand *et al.*, 1978; Whitehead & Hughes, 1975): (i) alteration from an epithelial to a fibroblastic type of growth pattern on first subculture; (ii) small eccentric nucleus; (iii) extensive cytoplasm; (iv) frilled and folded cell margins; (v) densely eosinophilic perinuclear zone; (vi) rapid removal of cells from plastic substrate following PBS wash and exposure to 0.25% trypsin, 0.004% versene; (vii) rapid adherence to plastic; (viii) cell suspensions easily converted to monodispersed cells without clumps; (ix) chromosome number of 46XX with no abnormalities. Further characterisation of MC cultures was carried out using immunohistochemical techniques. Putative MC were stained with a panel of monoclonal antibodies, using a standard indirect immunoperoxidase technique. Antibodies which were used included HMFG2 (Unipath), anti-vimentin and anti-keratin (Amersham International plc). In this study, cultured MC did not stain with HMFG2 (Wilson *et al.*, 1987), although there are varied reports in the literature which describe positive staining of some benign MC in wax-embedded tissue (Ghosh *et al.*, 1987) and negative staining in wet-fixed smears (Epenetos *et al.*, 1982). MC used in this study stained positively for both vimentin and keratin, as has been previously reported (Connell & Rheinwald, 1983) whereas epithelial cells were, until recently, believed to express only keratin. However, co-expression of both types of intermediate filaments has been reported in ovarian carcinomas (Veda *et al.*, 1987), and vimentin staining has not therefore been taken as definitive proof of MC without other confirmatory evidence. MC cultures were initially maintained on GM supplemented with 10% fetal calf serum. Cultures were passaged weekly using a 1:3 split ratio and cells from confluent cultures at passage 2–4 were used for most experiments. In the latter part of the study MC were maintained on GM supplemented with EGF (5 ng ml$^{-1}$) and hydrocortisone (0.4 $\mu$g ml$^{-1}$) and cells were then used up to passage 7. Fibroblasts from a variety of sources including malignant tumours, benign tumours, normal endometrium and a cell line CCD-14SK from the skin of a 34-year-old normal female (American Cell Collection) were also used as feeder cells.

*Soft agar assay*

Bases of 1 ml GM in 0.5% agar (Agar Noble) were prepared in 35 mm petri dishes (Nunc) and allowed to solidify at 4°C for 10 min. Feeder cells were added as a single cell suspension in a 1 ml layer of GM in 0.5% agar. After solidification of the middle layer, tumour cells were added as a single cell suspension in a 1 ml layer of GM in 0.3% agar. Control plates contained a middle layer consisting of medium and agar only. Cell numbers used varied according to the experiment and will be detailed in appropriate sections. Four dishes per test condition were routinely plated and incubation was carried out at 37°C in an humidified atmosphere of 95% air/5% $CO_2$. Colonies (>50 cells) were counted after 10–14 days, using an inverted microscope set at ×100 magnification.

*Mesothelial cell: tumour cell ratio*

A series of plates were prepared containing standard 1 ml base layers overlaid with $10^1$, $10^2$, $10^3$, $10^4$, $10^5$ and $10^6$ MC in 0.5% agar in GM. Tumour cells were overlaid in a separate top layer at $10^3$, $10^4$ and $10^5$ cells per dish in 0.3% agar in GM over each inoculum of MC or over a middle layer of GM in 0.5% agar. Dishes were scored for colonies after 7–10 days incubation at 37°C.

*Conditioned medium (CM)*

Growth medium which had been in contact with a confluent monolayer of MC for 3–5 days was used instead of MC in a soft agar assay. The middle MC-containing layer was omitted and CM was used undiluted in the top layer containing the tumour cells. The base layer contained GM, and CM was therefore present at a final dilution of 50%.

*Colony stimulating activity of cell-free ascites and serum from the same patient*

The colony stimulating activity (CSA) of cell-free ascites for TC has been described by others (Uitendaal *et al.*, 1983; Broxterman *et al.*, 1987). The supposition that preferential growth of ovarian cancer in the coelomic cavities is due to locally produced growth factors implies that there should be a difference in the growth promoting properties of serum and ascites, and paired samples of serum and ascites from the same patient were therefore tested for CSA, using OAW 42 as target cells. Aliquots of serum and ascites were clarified by centrifugation and used in the top tumour cell layer, as described for CM (see above).

*Increasing distance between feeder cells and tumour cells*

A series of plates was set up containing four separate layers of soft agar. TC (OAW 42) and MC or fibroblasts (CCD-14SK) were added at $10^4$ cells ml$^{-1}$ and $10^5$ cells ml$^{-1}$ respectively (10MC:1TC) such that feeder cells and TC were present in the same layer, adjacent layers or separated by one or two layers of soft agar. Each layer was allowed to solidify for 5 min at 4°C before addition of the next.

*Effect of epidermal growth factor (EGF) on the feeder effect of mesothelial cells*

EGF (Gibco) and hydrocortisone (Sigma) were added to GM to give final concentrations of 5 ng ml$^{-1}$ and 0.4 $\mu$g ml$^{-1}$ respectively. This supplemented medium was used as a diluent in the MC-containing middle layer. Control plates contained the supplemented middle layer without MC. The final concentration of EGF in the three-layer assay system was therefore 1.7 ng ml$^{-1}$.

### Results

*Ratio dependence*

Preliminary results clearly showed that MC promoted the clonogenic growth of OAW 42 cells in soft agar, and that the effect was strongly ratio dependent.

Results displayed in Figure 1 show that when TC (OAW 42) were in excess (10,000:1, 1,000:1, 100:1, 10:1) MC inhibited their clonogenic growth, but as the ratio approached 1:1 stimulation of clonogenic growth occurred which increased up to the maximum ratio tested (1,000 MC:1 TC). Significant inhibition of TC clonogenicity was seen at ratios of 1:100 and 1:10 MC:TC using TC innocula of $10^3$ and $10^4$, and at 1:1,000 and 1:100 MC:TC using a TC innoculum of $10^5$. Significant stimulation of the clonogenicity of $10^3$ TC was seen at 100:1 and 1,000:1, of $10^4$ TC at 1:1, 10:1 and 100:1 and of $10^5$ TC at 1:1 and 10:1. No significant differences were found at ratios of 1:1 and 10:1 (TC = $10^3$), 1:1,000 (TC = $10^4$), and 1:10,000 and 1:10 (TC = $10^5$). Inhibition and stimulation were therefore depen-

**Figure 1** The effect of different ratios of mesothelial cells and tumour cells on the plating efficiency of OAW 42 in soft agar. ■ = $10^5$TC; ○ = $10^4$TC; ● = $10^3$TC; Control colony number (−MC) $10^3$ = 13.2 ± 8.4 ($n$ = 4); $10^4$ = 79.2 ± 22.8; $10^5$ = 709.2 ± 40. Standard deviations were of a similar order of magnitude for the other points plotted. The points at which significant inhibition and stimulation occurred are detailed in the text. Similar results were obtained in three experiments.

dent on the ratios of the two cell types rather than absolute numbers, since both were seen at all three densities of TC. Similar results were obtained in three experiments.

*Ovarian tumour cell lines*

Results obtained with three other ovarian TC lines showed that not all TC populations were responsive to the feeder effect of MC (10MC:1TC). OAW 28 (P15) had a plating efficiency of 0.02% in the absence of MC which increased to 3.2% in their presence. However, 32M (P2–P13) and A7 (P82) were non-clonogenic in soft agar and did not form colonies in the presence of MC.

*Primary ovarian tumour cultures*

Comparison of early (P26) and late (P102) cultures of OAW 42 revealed differences in the responsiveness of TC relating to passage level (Table I). Cells from P26 were responsive but the magnitude of the effect was considerably less than that obtained with P102. Several primary cultures were therefore tested to ensure that the phenomenon was not an artefact relating to long-term culture. Results for seven primary cultures are shown in Table II. Four cultures which formed colonies in soft agar responded to MC with an increase in plating efficiency (11M, ×2.4; 1B, ×14; 25M, ×2.3; 31M, ×5.3) but three others which were non-clonogenic remained non-clonogenic in the presence of MC (1S, 59M, 41M). 59M and 41M were subsequently developed into continuous cell lines.

*Conditioned medium (CM)*

Addition of CM to soft agar cultures of OAW 42 significantly increased its plating efficiency in three separate experiments but the magnitude of the increase was considerably less than that achieved when intact viable MC were included in the soft agar (Table III).

*CSA of cell-free ascites and serum from the same patient*

Pairs of serum/ascitic fluid taken at the same time from seven patients were tested for CSA against OAW 42. Results outlined in Table IV show that differences did exist between serum and ascities. Only 1/7 of the serum samples tested showed significantly greater activity than the corresponding ascitic fluid sample (81D; $P \leqslant 0.005$). For the other pairs there was a significant decrease in CSA between serum and ascites in 5/6 pairs. However, cells grown with the human serum still showed significantly greater clonogenicity than those grown with GM in 3/7 pairs, similar clonogenicity in 2/7 pairs and significantly reduced clonogenicity in 2/7 pairs.

**Table I** Comparison of low and high passage numbers of OAW 42 for responsiveness to mesothelial cells

| | $-MC$ | $+MC$ |
|---|---|---|
| P26 | 0.22±0.02[a] | 0.46±0.1 |
| | | 0.50±0.2 |
| P102 | 1.30±0.2 | 13.00±1.4 |
| | | 13.80±1.4 |

[a]Plating efficiency (%)±s.d.

Tumour cells were plated at $10^4$ per dish and MC at $10^5$ per dish. MC from two patients were tested.

*Increasing distance between MC and TC*

The effect of increasing distance between target cell and feeder cell was determined on three occasions using 58MC and on three occasions using CCD-14SK at a ratio of 10 feeders:1TC. Results are shown in Table V for two experiments in which 58MC and CCD-14SK were set up at the same time using the same passage of OAW 42. The maximum feeder effect with both types of feeder cell was obtained when feeder cell and target cell were present in the same layer, although CCD-14SK were significantly less effective than 58MC. There was a gradual reduction in feeder effect as distance increased such that when two layers of agar were interspersed between the cell populations the feeder effect of 58MC was 50–52% of the maximum effect obtained, contrasting with 39–41% with CCD-13SK. In the two other experiments (data not shown) there were similar results. When all results obtained were combined a reduction of 55±8% was obtained for 58MC and of 27±22% for CCD-14SK; these values were significantly different ($P < 0.05$; Student's $t$ test). The gross morphology of the TC colonies also varied according to the relative positions of MC and TC. When the two cell types were mixed TC colonies were predominantly solid, containing small, densely packed cells. As distance increased the number of cystic colonies increased, and when MC were absent the colonies were entirely cystic. A total of 100 colonies were scored as dense, loose refractile or cystic, and these results are shown as histograms in Figure 2. The microscopic appearance of the three colony types are shown in Figures 3 and 4.

*Specificity of effect*

A number of non-ovarian TC lines were tested for responsiveness to the feeder effect of MC. The response was variable as was found with ovarian carcinoma (Table VI) with inhibition, stimulation and no effect observed in different cell lines. FL (transformed amnion), PA1 (teratoma), HT29 (colorectal carcinoma) and Go-GIJKT (astrocytoma) all showed enhanced clonogenicity in the presence of MC; ZR75 (breast carcinoma), BeWo (choriocarcinoma), TR126 (cheek carcinoma) and CCM (astrocytoma) were all unresponsive; and T24 (bladder carcinoma) showed reduced clonogenicity in the presence of MC.

Feeder cells from a variety of sources were also tested for their ability to promote the clonogenic growth of TC. These included MC from cancer patients ($n=13$), fibroblasts from benign tumours ($n=2$) and malignant tumours ($n=1$), as well as normal fibroblasts from endometria obtained at hysterectomy ($n=2$), fibroblasts from a follicular cyst of pregnancy ($n=1$) and a fibroblast cell line (CCD-14SK) from the skin of a 34-year-old female. Results are shown in Table

**Table II** Response of primary ovarian tumour cell cultures to feeder effect of mesothelial cells

| *Culture* | *TC no.* | *MC:TC* | $-MC$ | $+MC$ | *Factor increase*[a] |
|---|---|---|---|---|---|
| 11M | $10^5$ | 2:1 | 0.0024±0.0015 | 0.006±0.001 | 2.4 |
| 1B | $10^4$ | 10:1 | 0.016±0.011 | 0.23±0.10 | 14.4 |
| 25M | $2\times10^4$ | 5:1 | 0.315±0.065 | 0.72±0.17 | 2.3 |
| 31M | $10^5$ | 5:1 | 0.034±0.004 | 0.179±0.067 | 5.3 |
| 1S | $10^4$ | 10:1 | 0 | 0 | 0 |
| 59M | $2\times10^4$ | 5:1 | 0 | 0 | 0 |
| 41M | $10^4$ | 10:1 | 0 | 0 | 0 |

[a]Factor increase is the plating efficiency in the presence of MC/plating efficiency in the absence of MC.

**Table III** The effect of conditioned medium on the clonogenic growth of OAW 42

| | *Control (C)* | *CM* | *CM:C* | *P* |
|---|---|---|---|---|
| OAW 42. P58 | 0.29±0.07%[a] | 0.44±0.07% | 1.52 | ≤0.005 |
| OAW 42. P87 | 4.20±0.30% | 5.70±1.05% | 1.36 | ≤0.05 |
| OAW 42. P80 | 0.09±0.03% | 0.42±0.06% | 4.67 | ≤0.001 |

[a]Plating efficiency ± s.d.
Conditioned medium which had been in contact with confluent monolayers of MC for 3–5 days was used in all three experiments.

**Table IV** Comparison of CSA of serum and ascites from the same patient, using OAW 42 as target cells

| | | | Test PE/control PE | |
|---|---|---|---|---|
| *No.* | *Ascitic fluid* | *Serum* | *Ascitic fluid* | *Serum* |
| 45D | 7.0±0.4[a] | 2.0±0.3 | 3.2 | 0.9 |
| 46D | 9.2±0.4 | 5.6±0.5 | 4.2 | 2.5 |
| 53D[b] | 10.8±1.1 | 0.2±0.06 | 4.9 | 0.09 |
| 64D | 10.7±1.4 | 7.8±3.5 | 4.9 | 3.5[c] |
| 79D | 8.1±1.8 | 2.6±0.6 | 3.7 | 1.2 |
| 80D | 3.1±0.2 | 0.3±0.08 | 1.4 | 0.14 |
| 81D | 4.3±0.5 | 6.6±0.53 | 1.9 | 3.0 |
| Control (GM) | 2.2±0.26 | | | |

[a]Plating efficiency (PE) ± s.d.; [b]53D was plasma; all other samples were serum; [c]Not significant; all other differences between serum and ascites were significant ($P<0.005$).

VII. Although fibroblasts from all except one of the endometria were able to stimulate TC growth the magnitude of the stimulation was less than that of MC. Using only the experiments in which feeder cells and target cells were present at a ratio of 10:1 and comparing MC from cancer patients ($n=10$) with fibroblasts from benign conditions ($n=5$), the fibroblasts showed a significantly lower stimulatory effect (MC=8.4±6.6; fibroblasts=2.64±1.49; $P\leqslant0.05$).

*Effect of EGF on feeder effect of MC*

The effect of EGF on the feeder effect of MC has been determined in four separate experiments using OAW 42 as target cells and in one experiment using 32M. Results are shown in Table VIII. OAW 42 showed a variable increase in plating efficiency with EGF (range 0.97–3.63, mean 2.2±1.2) but in all four experiments the feeder effect of MC was reduced in the presence of EGF. Taking into account the enhanced clonogeneicity of the tumour cells in the presence of EGF, the magnitude of the reduction in feeder effect ranged from 1.04 to 12.14. The MC were also variably responsive to EGF and there was a significant relationship between the magnitude of the reduction in feeder effect and the response of MC to EGF ($r=0.906$, $P\leqslant0.01$), as evidenced by the increase in clonogenicity of MC in the presence of EGF. There was also a significant relationship between the control colony number of MC (i.e. in the absence of EGF) and the magnitude of the stimulatory effect of MC ($r=0.712$, $P\leqslant0.05$). It should be noted here that when these experiments were done MC were routinely maintained on GM supplemented with EGF and hydrocortisone. Under these growth conditions some colony formation occurred in soft agar even when the GM contained neither growth factor. The morphology of the colonies was distinctly different from TC colonies and since they were also present in a different layer from the TC, there was no possibility of confusion during colony counting.

The results obtained when 32M was used as target differed in that these cells were unresponsive to MC but formed small clusters (6–12 cells) in the presence of EGF. Interestingly, in spite of their unresponsiveness to MC, their response to EGF was still reduced in the presence of MC.

### Discussion

The ability of fibroblasts to promote the growth of TC and normal epithelium is well documented (Stanley & Parkinson, 1979; Taylor-Papadimitriou *et al.*, 1977; Peehl & Ham, 1980; Hamburger *et al.*, 1981; Brattain *et al.*, 1982; Citron *et al.*, 1986; Gallie *et al.*, 1982; Kirk *et al.*, 1981, 1983; Koopman & Cotton, 1984; Laboisse *et al.*, 1981; Rheinwald & Beckett, 1981; Rosenstraus *et al.*, 1984) but this is the first report on the feeder effect of MC for human ovarian cancer. It was originally hypothesised that locally produced factors could contribute to the restricted pattern of spread of ovarian cancer and the finding that MC can stimulate the clonogenic growth of ovarian TC supports this idea. Ascitic fluids from cancer patients also possess colony stimulating activity for some TC (Uitendaal *et al.*, 1983; Broxterman *et al.*, 1987) and this may be due to conditioning of the fluid by MC and/or TC. Although human serum/plasma also promoted clonogenic growth of TC above control levels (GM) in 3/7 samples tested, it was interesting to observe that the colony stimulating activity of serum was appreciably lower than ascitic fluid from the same patient in 5/7 pairs tested. This finding, together with the relative inactivity of conditioned medium compared with the continuous presence of viable cells, suggests that locally produced factors can stimulate clonogenic growth of ovarian TC and that these factors may not reach the systemic circulation in concentrations which are high enough to promote clonogenic growth. This is also suggested by the finding that increasing distance between MC and TC reduces the feeder effect. Although fibroblasts also stimulated the clonogenic growth of TC, this effect was significantly less than that obtained with MC. The observation that colony morphology changed depending on the distance between feeder cell and tumour cell was interesting. The cystic colonies represent a differentiated function of the epithelial tumour cells, arising due to fluid transport. It is possible that the dense colonies reflect a lack of differentiation since it has been shown that fibroblast feeder layers can inhibit the differentiation of retinoic acid-treated embryonal carcinoma cells (Rosenstraus *et al.*, 1984). Even though the fibroblast is ubiquitously distributed in the body, a metastasising TC is unlikely to encounter fibroblasts in the same

**Table V** Effect of increasing the distance between feeder cells and tumour cells on the plating efficiency of tumour cells

| *Test* | *58MC* | *CCD-14SK* | *58MC* | *CCD-14SK* |
|---|---|---|---|---|
| 1 | 8.64±2.32(100)[a] | 3.52±0.20(100) | 20.08±1.84(100) | 11.12±1.0(100) |
| 2 | 5.28±0.96(61) | 2.32±0.40(66) | 16.88±1.52(84) | 6.36±0.64(57) |
| 3 | 3.68±0.12(42) | 1.32±0.24(37) | 12.32±2.12(61) | 4.92±0.52(44) |
| 4 | 4.48±0.44(52) | 1.36±0.24(39) | 10.04±1.64(50) | 4.56±0.92(41) |
| 5 | 0.10±0.08 | 0.08±0.04 | 4.16±0.88 | 4.04±0.88 |

Feeder cells and OAW 42 cells were plated at a ratio of 10:1. Results are shown as PE ± s.d.
[a]Plating efficiency expressed in parentheses as a percentage of 1, i.e. maximum stimulation.
1, MC and TC in same layer; 2, MC and TC in adjacent layers; 3, MC and TC separated by one layer; 4, MC and TC separated by two layers; 5, TC only.

**Figure 2** The distribution of different colony types of OAW 42 obtained with varying distances between TC and MC. (**a**) dense colonies; (**b**) loose refractile colonies; (**c**) cystic colonies. The dotted lines show the colony distribution obtained in the presence of fibroblasts. There were no dense colonies in conditions 2, 3, 4 and 5 when fibroblasts were used. 1, MC and TC in same layer; 2, adjacent layers; 3, separated by one layer; 4, separated by two layers; 5, no MC.

**Figure 3** Phase contrast photomicrographs of the different colony types. (**a**) C=cystic; LR=loose refractile: (**b**) dense colony. Magnification ×82.5.

**Table VI** Responsiveness of non-ovarian carcinoma cell lines to the feeder effect of mesothelial cells

| *Cell line* | *Tumour type* | *−MC* | *+MC* |
|---|---|---|---|
| ZR 75 | Breast carcinoma | 0.08±0.0[a] | 0.08±0.06 |
| FL | Transformed amnion | 1.50±0.28 | 6.70±0.60 |
| PA-1 | Teratoma | 0 | 1.10±0.20 |
| BeWo | Choriocarcinoma | 0 | 0 |
| HT29 | Colorectal carcinoma | 0.07±0.03 | 5.56±0.22 |
| TR126 | Cheek carcinoma | 0 | 0 |
| Go-GIJKT | Astrocytoma | 0.20±0.19 | 9.40±1.36 |
| CCM | Astrocytoma | 0 | 0 |
| T24 | Bladder carcinoma | 3.10±0.33 | 0.66±0.22 |

[a]Plating efficiency ± s.d.

All lines were tested at a ratio of 10MC:1TC, with the exception of T24, which was tested at a ratio of 5:1.

**Figure 4** H & E stained preparations of the different colony types. (**a**) cystic; (**b**) dense. Magnification ×330.

**Table VII** The effect of different feeder cell populations on the clonogenicity of OAW 42

| | | | *PE* | | |
|---|---|---|---|---|---|
| *Feeders* | *Source* | *Ratio*[a] | *−MC* | *+MC* | *Increased*[b] |
| *Mesothelial cells* | | | | | |
| 37M | Ca.Ov | 10:1 | 0.2±0.05 | 3.4±0.5 | 17 |
| 38M | Ca.Ov | 10:1 | 0.2±0.05 | 4.4±0.8 | 22 |
| 40M | Ca.Ov | 10:1 | 1.3±0.20 | 13.8±1.4 | 10.6 |
| 42M | Ca.Ov | 10:1 | 1.3±0.20 | 13.0±1.4 | 10 |
| 20 | Ca.Ov | 10:1 | 0.4 | 0.8±0.02 | 2 |
| 58 | Ca.Ov | 10:1 | 1.2±0.10 | 5.9±1.3 | 4.9 |
| 53 | Ca.Ov | 10:1 | 1.8±0.40 | 14.2±2.4 | 7.9 |
| 18 | Meso | 10:1 | 0.4 | 0.8±0.02 | 2 |
| 35 | Ca.Br | 10:1 | 0.4±0.03 | 3.1±0.5 | 7.7 |
| 28 | Ca.Ov | 2:1 | 0.4±0.09 | 1.6±0.1 | 4 |
| 2 | Ca.Ov | 2:1 | 0.6±0.03 | 1.2 | 2 |
| 4 | Ca.Ov | 5:1 | 0.6±0.03 | 2.2 | 3.7 |
| 41M | Ca.Ov | 5:1 | 0.6±0.03 | 2.2 | 3.7 |
| 5Om | Bn.Ov | 10:1 | 0.04±0.03 | 4.2±0.6 | 105 |
| *Fibroblasts* | | | | | |
| E1 | Emdo. | 10:1 | 1.2±0.05 | 0.6±0.2 | 0.5 |
| E2 | Endo. | 10:1 | 1.3±0.20 | 4.4±0.2 | 3.4 |
| CCD-14sk | Skin | 10:1 | 1.5±0.60 | 6.2±1.2 | 4.1 |
| 5 | Bn.Ov. | 10:1 | 3.1±0.50 | 11.0±1.1 | 3.5 |
| 19 | Bn.Ov | 10:1 | 3.1±0.50 | 5.2 | 1.7 |
| 3 | F.C.P. | 5:1 | 0.6±0.003 | 0.94 | 1.6 |
| 41 | Ca.Ov | 10:1 | 1.5±0.60 | 9.9±0.7 | 6.6 |

[a]MC:TC; [b]PE in presence of MC/PE in absence of MC. Ca.Ov, carcinoma of ovary; Meso, mesothelioma; Ca.Br, carcinoma of breast; Om, omentum; Bn.Ov, benign tumour of ovary; Endo, endometrium; FCP, follicular cyst of pregnancy.

numbers as those of MC in the coelomic cavity and an excess of feeder cells is obviously necessary for stimulation. It is therefore suggested that the coelomic cavity can provide a uniquely favourable environment for TC growth, in that mesothelial cells are present in large numbers. The generally restricted spread of ovarian cancer beyond the confines of the coelomic cavity and the failure of TC which reach the

**Table VIII** Influence of EGF on the feeder effect of mesothelial cells

| | *PE of TC* | | *PE of MC* | |
|---|---|---|---|---|
| | *−EGF* | *+EGF* | *−EGF* | *+EGF* |
| 1 −mc | 2.36±0.42 | | | |
| +mc | 4.98±0.20(2.1)[a] | 2.62±0.73(1.1)[b] | 0.315±0.105 | 0.658±0.257(2.1)[c] |
| 2 −mc | 0.54±0.06 | 1.96±0.25 | | |
| +mc | 3.51±0.36(6.5) | 2.14±0.26(1.1) | 0.083±0.007 | 0.215±0.017(2.6) |
| 3 −mc | 0.45±0.01 | 1.29±0.23 | | |
| +mc | 5.49±0.27(12.2) | 4.95±0.16(3.8) | 0.043±0.001 | 2.200±0.377(52) |
| 4 −mc | 0.53±0.05 | 1.76±0.08 | | |
| +mc | 19.07±1.24(36) | 9.23±0.68(12.1) | 0±0 | 0.700±0.087(700) |
| 5 −mc | 0±0 | 1.33±0.16 | | |
| +mc | 0±0(0) | 0.73±0.19(0.5) | 0.409±0.054 | 1.123±0.258(2.7) |

[a]Relative increase in PE in absence of EGF; [b]Relative increase in PE in presence of EGF; [c]Relative increase in PE of MC in presence of EGF; MC and TC colonies could be counted separately because they were of differing morphology and in different focal planes.

EGF was present at a final concentration of 1.7 ng ml$^{-1}$.

Experiments 1–4 used OAW 42 as target cells.

Experiment 5 used 32M as target cells.

systemic circulation via peritoneovenous shunts to proliferate (Tarin *et al.*, 1984) may reflect this requirement.

Mechanisms underlying the epithelial–mesenchymal interactions have not been elucidated and no clear picture emerges from a study of the literature. Variable factors have included source of feeder cell, type of target cell, method of culture, treatment of feeder cell and type of medium. The general consensus appears to be: (i) that fibroblasts from any source including normal adult, neonatal, tumour-derived, embryonic and non-human can stimulate epithelial cell growth; (ii) that a variety of normal and transformed cell types can respond to the feeder effect; (iii) that co-cultivation of feeder cells and epithelial cells gives considerably better growth than does the use of conditioned medium. Contradictions to these general conclusions may be found, however. Kirk *et al.* (1981, 1983) concluded that the feeder effect was dependent on non-proliferation of feeder cells since monolayers of feeders were inhibitory whereas agar-suspended feeders stimulated the growth of target cells. These authors also found differences in the inhibitory behaviour of fibroblasts from different sources. Thus, neonatal human lung fibroblasts were inhibitory in monolayer, kidney tumour derived fibroblasts were stimulatory and fibroblasts from the skin of a Down's syndrome neonate were both inhibitory and stimulatory depending on the ratio of feeder cell to target cell (Kirk *et al.*, 1983). Other authors have found no differences between fibroblasts from different sources (Taylor-Papadimitriou *et al.*, 1981; Gallie *et al.*, 1982), while in the present study only one (endometrium derived fibroblasts) of fourteen feeder cell populations failed to enhance tumour growth. Although Kirk *et al.* (1983) reported that mitomycin C treatment of monolayers abolished their inhibitory effect, other workers have found that untreated monolayers could enhance tumour growth (Brattain *et al.*, 1982; Gallie *et al.*, 1982). Results from the present study suggest that variability between difference studies may be due to a combination of differences in feeder cell to target cell ratios and the distance between feeder cell and target cell. In the two studies where monolayers and target cells were separated by a layer of agar either no stimulation of target cell growth (Hamburger *et al.*, 1981) or inhibition of target cell growth (Kirk *et al.*, 1983) was found. This idea is supported by the finding that feeder effect decreased with increasing distance between feeder cell and target cell.

The finding that EGF reduced the feeder effect of MC contrasts with the results of other workers showing an enhancement of the feeder effect in the presence of EGF (Rheinwald & Green, 1977; Stanley & Parkinson, 1979; Taylor-Papadimitriou *et al.*, 1977; Peehl & Ham, 1980; Hamburger *et al.*, 1981). In all these studies the feeder cells were either irradiated or treated with mitomycin C. The dual finding of significant correlations between: (i) response of MC to EGF and reduction in feeder effect and (ii) colony formation by MC in the absence of EGF and magnitude of the feeder effect, provides strong evidence for an association between proliferation and feeder effect, as previously reported by Kirk *et al.* (1981, 1983).

It would appear that the effector molecule may be inhibitory or stimulatory depending on the target cell and the relative numbers of MC and TC. 32M was unresponsive to MC but had a reduced response to EGF in their presence. While this may have been due to reduced availability of EGF because of binding by MC, it could also have been due to inhibition of 32M by MC, a phenomenon which could only be observed when the cells were actually stimulated to proliferate by EGF. Further support for the idea of a dual role for the effector molecule comes from the marked inhibition of clonogenicity of T24 in the presence of MC. Feeder effect was also inhibitory when TC were present in excess of MC.

Whether any of these interactions are of relevance *in vivo* cannot be directly concluded from these results but they do provide indirect evidence for the view that epithelial–mesenchymal interactions within the coelomic cavity may be important in influencing the pattern of spread of ovarian cancer.

The work was supported by grants from the Medical Research Council and South Manchester Regional Health Authority, and was carried out in the Department of Obstetrics and Gynaecology at Withington Hospital, South Manchester and the Department of Reproductive Pathology, St Mary's Hospital, Manchester. The support and accommodation provided by Professors M. Elstein and H. Fox in these respective laboratories is gratefully acknowledged, as is that of Dr P.R. Golding, Medical Director, Oncology Research Lboratory, Derby City Hospital, where the work was completed. The ovarian tumour cell line A7 was kindly supplied by Dr G. Roos, University Hospital, Umea, Sweden. CCM and Go-GIJKT were kindly supplied by Dr R.I. Freshney, Oncology Research Laboratory, Glasgow. PA1 and BeWo were obtained from the American Cell Collection. The expert technical help of Mrs C.M. Taylor in Manchester and Mrs M. Dent in Derby is also gratefully acknowledged, as is Miss H. Lee's assistance with the preparation and typing of the manuscript.

### References

ABU SINNA, G., BECKMAN, G., LUNDGREN, E., NORDENSON, I. & ROOS, G. (1979). Characterization of two new human ovarian carcinoma cell lines. *Gynaecol. Oncol.*, **7**, 267.

BRATTAIN, M.G., BRATTAIN, D.E., SARRIF, A.M., McRAE, L.J., FINE, W.D. & HAWKINS, J.G. (1982). Enhancement of growth of human colon tumour cell lines by feeder layers of murine fibroblasts. *J. Natl Cancer Inst.*, **69**, 767.

BROXTERMAN, H.J., SPRENKELS-SCHOTTE, C., ENGELEN, PH., LEYVA, A. & PINEDO, H.M. (1987). Analysis of human ascites effect on clonogenic growth of human tumour cell lines and NRK-49F cells in soft agar. *Int. J. Cell Cloning*, **5**, 158.

BUICK, R.N., FRY, S.E. & SALMON, S.E. (1980). Effect of host cell interactions on clonogenic carcinoma cells in human malignant effusions. *Br. J. Cancer*, **41**, 695.

CITRON, M.L., JAFFE, N.D., HAMBURGER, A.W. and 5 others (1986). Improvement of human tumor cloning assay by suspension of fibroblasts into the bottom layer of agarose. *Cancer*, **57**, 2357.

CONNELL, N.D. & RHEINWALD, J.G. (1983). Regulation of the cytoskeleton in mesothelial cells: reversible loss of keratin and increase in vimentin during rapid growth in culture. *Cell*, **34**, 245.

EPENETOS, A.A., CANTI, G., TAYLOR-PAPADIMITRIOU, J., CURLING, M. & BODMER, W.F. (1982). Use of two epithelium-specific monoclonal antibodies for diagnosis of malignancy in serous effusions. *Lancet*, **ii**, 1004.

GALLIE, B.L., HOLMES, W. & PHILLIPS, R.A. (1982), Reproducible growth in tissue culture of retinoblastoma tumor specimens. *Cancer Res.*, **42**, 301.

GHOSH, A.K., GATTER, K.C., DUNNILL, M.S. & MASON, D.Y. (1987). Immunohistological staining of reactive mesothelium, mesothelioma and lung carcinoma with a panel of monoclonal antibodies. *J. Clin. Pathol.*, **40**, 19.

HAMBURGER, A.W., WHITE, C.P. & BROWN, R.W. (1981). Effect of epidermal growth factor on proliferation of human tumor cells in soft agar. *J. Natl Cancer Inst.*, **67**, 825.

HAMBURGER, A.W., WHITE, C.P., LURIE, K. & KAPLAN, R. (1986). Monocyte-derived growth factors for human tumor clonogenic cells. *J. Leuk. Biol.*, **40**, 381.

KIRK, D., SZALAY, M.F. & KAIGHN, M.E. (1981). Modulation of growth of a human prostatic cancer cell line (PC-3) in agar culture by normal human lung fibroblasts. *Cancer Res.*, **41**, 1100.

KIRK, D., KAGAWA, S. & VENER, G. (1983). Comparable growth regulation of five human tumor cell lines by neonatal human lung fibroblasts in semisolid culture media. *Cancer Res.*, **43**, 3754.

KOOPMAN, P., & COTTON, R.G.H. (1984). A factor produced by feeder cells which inhibits embryonal carcinoma cell differentiation. Characterization and partial purification. *Exp. Cell Res.*, **154**, 233.

LABOISSE, C.L., AUGERON, C. & POTET, F. (1981). Growth and differentiation of human gastrointestinal adenocarcinoma stem cells in soft agarose. *Cancer Res.*, **41**, 310.

MOURIQUAND, J., MOURIQUAND, C., PETITPAS, E. & MERMET, M.A. (1978). Long term tissue culture of human pleural effusions: a cytological follow-up. *In Vitro*, **14**, 591.

PEEHL, D.M. & HAM, R.G. (1980). Growth and differentiation of human keratinocytes without a feeder layer or conditioned medium. *In Vitro*, **16**, 516.

RHEINWALD, J.G. & BECKETT, M.A. (1981). Tumorigenic keratinocyte lines requiring anchorage and fibroblast support cultured from human squamous cell carcinomas. *Cancer Res.*, **41**, 1657.

RHEINWALD, J.G. & GREEN, H. (1977). Epidermal growth factor and the multiplication of cultured human epidermal keratinocytes. *Nature*, **265**, 421.

ROSENSTRAUS, M.J., STERMAN, B., CARR, A. & BRAND, L. (1984). Fibroblast feeder layers inhibit differentiation of retinoic acid-treated embryonal carcinoma cells by increasing the probability of stem cell renewal. *Exp. Cell Res.*, **152**, 378.

STANLEY, M.A. & PARKINSON, E.K. (1979). Growth requirements of human cervical epithelial cells in culture. *Int. J. Cancer*, **24**, 407.

TARIN, D., PRICE, J.E., KETTLEWELL, M.G.W., SOUTER, R.G., VASS, A.C.R. & CROSSLEY, B. (1984). Mechanisms of human tumor metastasis studied in patients with peritoneovenous shunts. *Cancer Res.*, **44**, 3584.

TAYLOR-PAPADIMITRIOU, J., SHEARER, M. & STOKER, M.G.P. (1977). Growth requirements of human mammary epithelial cells in culture. *Int. J. Cancer*, **20**, 903.

TAYLOR-PAPADIMITRIOU, J., PETERSON, J.A., ARKLIE, BURCHELL, J., CERIANI, R.L. & BODMER, W.F. (1981). Monoclonal antibodies to epithelium-specific components of the human milk fat globule membrane: production and reaction with cells in culture. *Int. J. Cancer*, **28**, 17.

UITENDAAL, M.P., HUBERS, H.A.J.M., McVIE, J.G. & PINEDO, H.M. (1983). Human tumour clonogenicity in agar is improved by cell-free ascites. *Br. J. Cancer*, **48**, 55.

VEDA, G., INOUE, M. and 5 others (1987). Expression of vimentin in common epithelial tumours of the ovary. *Acta Obstet. Gynaecol. Jpn.* **39**, 1151.

WARD, B.G., LOWE, D.G. & SHEPHERD, J.H. (1987). Patterns of expression of a tumour-associated antigen, defined by the monoclonal antibody HMFG2, in human epithelial ovarian carcinoma. *Cancer*, **60**, 787.

WELANDER, C.E., NATALE, R.B. & LEWIS, J.L. (1982). *In vitro* growth stimulation of human ovarian cancer cells by xenogeneic peritoneal macrophages. *J. Natl Cancer Inst.*, **69**, 1039.

WHITEHEAD, R.H. & HUGHES, L.E. (1975). Tissue culture studies of malignant effusions. *Br. J. Cancer*, **32**, 512.

WILSON, A.P. (1984). Characterization of a cell line derived from the ascites of a patient with papillary serous cystadenocarcinoma of the ovary. *J. Natl Cancer Inst.*, **72**, 513.

WILSON, A.P., FORD, C.H.J., NEWMAN, C.E. & HOWELL, A. (1987). Cis-platinum and ovarian carcinoma. *In vitro* chemosensitivity of cultured tumour cells from patients receiving high dose cis-platinum as first line treatment. *Br. J. Cancer*, **56**, 763.